# Tracking Tool for Fiddler Crabs in Natural Settings to Promote a Model Organism for Synchrony

**DOI:** 10.1111/nyas.70213

**Published:** 2026-03-24

**Authors:** Hiba Khatib, Daniel M. Abrams, Guy Amichay

**Affiliations:** ^1^ Computer Science Northwestern University Evanston Illinois USA; ^2^ Engineering Sciences and Applied Mathematics Northwestern University Evanston Illinois USA; ^3^ Northwestern Institute on Complex Systems Northwestern University Evanston Illinois USA; ^4^ National Institute for Theory and Mathematics in Biology Northwestern University Chicago Illinois USA; ^5^ Physics and Astronomy Northwestern University Evanston Illinois USA

**Keywords:** animal tracking, fiddler crabs, lek, synchrony, wildlife

## Abstract

Synchronization has been studied across vastly different spatiotemporal scales in physical, biological, and social systems. Research into the mechanisms that give rise to temporal order, however, has been, by and large, mostly theoretical. Here, we seek to advance the fiddler crab as a model organism for collective synchronization. To attract mates, males of many fiddler crab species wave their claws in sync. These crabs are found in many places around the world, so they are generally accessible and observable. Translating observation (e.g., from recorded video) into actionable data, however, remains a challenge. We provide an easy‐to‐use and open source tracking algorithm that detects claw wave activity in video recordings and preserves IDs over time. We demonstrate the robustness of the algorithm by running it on videos displaying different species from different locations. We discuss possible future directions (both in the lab and the field) and call for renewed interest in these unique animals.

## Graphical abstract

We introduce an easy‐to‐use and open source tracking algorithm that detects claw waves in field video recordings and preserves IDs over time to advance the widely found fiddler crabs as a model organism for collective synchronization. This software translates video observations into actionable data to advance the study of synchronization beyond theory. We demonstrate the robustness of the algorithm by running it on videos of different species and in different locations.

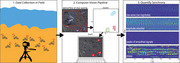

## 1 Introduction

Synchronization is all around us, yet the study of natural biological systems that synchronize—regardless of scale—is relatively lacking.^1^ Perhaps one reason for this is the difficulty in obtaining precise measurements. Quantifying neural activity in a brain poses both technical as well as ethical considerations [1]. Alternatively, measuring, for instance, synchronous fireflies presents different difficulties, due to their limited abundance [2]. Here, we wish to suggest a somewhat overlooked example: the concerted claw waving of fiddler crabs. Fiddler crabs are small crustaceans that live in salt marshes, mangroves, beaches, and mudflats; during their mating season, males wave their claws together in an attempt to draw the attention of the females. Note that fiddler crabs have been studied extensively, see, for example, [3, 4, 5, 6, 7, 8, 9], though these studies focus primarily on other aspects of crab behavior, not necessarily their synchrony.

Fiddler crabs are found on every continent except Antarctica [10], making them easily accessible. There are still many open questions regarding their behavior, most notably, *why*, and *how*, they synchronize. Multiple hypotheses exist, but conclusive evidence supporting one particular theory is yet to be found. Due to their robust nature, there is potential for experimental manipulation, lab studies, or even neural recordings. But to start off, the first hurdle is measuring their behavior in their natural habitat. Here, we offer a novel algorithm that can reliably track their waving patterns while preserving IDs over time (i.e., knowing who is whom). In other words, we offer an algorithm that enables us to reconstruct the interaction network of the group, and to observe and quantify their dynamics.

Tracking groups of animals is not a new endeavor. Many commonly used animal tracking tools have made it easier to analyze behavior in lab settings, but they often perform poorly when used outdoors in natural environments. ToxTrac, for example, is a program that can track multiple unmarked animals in 2D and 3D using background subtraction and blob detection. However, it assumes a clean and stable background with good lighting conditions that are usually only found in lab tanks or arenas [11]. In the field, where shadows, moving plants, and changes in lighting are common, this kind of method struggles to separate real movement from noise. Tracktor is a Python‐based tool that uses thresholding and size filters to detect objects [12]. It is flexible and works well when animals are clearly visible against a simple background, but like ToxTrac, it has trouble in chaotic environments where animals are camouflaged or where the background is cluttered. Another existing tool, idTracker, creates a fingerprint for each animal based on its appearance, allowing it to keep track of individuals even when they overlap or briefly disappear [13]. However, it relies heavily on high‐quality, consistent video. All three tools were built with a focus on lab conditions and tend to fall short when applied to complex, real‐world videos with many animals and objects moving in unpredictable ways.

DeepLabCut is one of the most popular modern tools for animal tracking and pose estimation. It uses deep learning to track specific body parts without the need for physical markers [14, 15]. By training a neural network based on DeeperCut [16, 17, 18, 19], a model originally designed for tracking human limbs, it can learn to recognize the body parts of an animal in several hundred labeled frames. This makes DeepLabCut accurate and flexible in lab studies where conditions are clean and controlled, but it fails when it comes to field recordings. The tool expects high‐resolution, stable footage with consistent lighting, and its accuracy drops in messy outdoor videos where animals are partially hidden, blend into the scenery, or move quickly across the frame. Moreover, even though it uses transfer learning to reduce the need for big data sets, researchers still have to manually label new frames for every new species, camera angle, or background [14]. This extra step makes DeepLabCut less practical for field studies, where conditions change frequently and labeled data are inaccessible.

Here, we describe a new method for tracking collective animal behavior in the wild. Our method is robust to variable field conditions and low‐resolution recordings, albeit applicable primarily to fiddler crabs. Note that we focus here on their synchronous behavior, though this method could presumably be useful for any claw waving activity [8].

## 2 Methods

### 2.1 Data Sets

**FIGURE 1:**
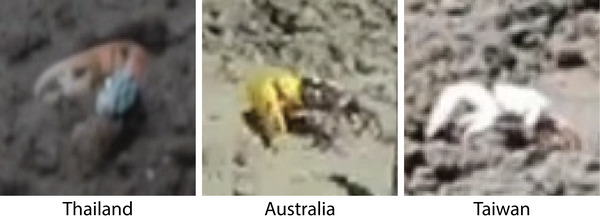
Fiddler crab species. Examples of fiddler crab diversity across field sites. Crabs from Laem Phak Bia, Thailand (*Austruca annulipes*), Darwin, Australia (*Austruca mjoebergi*), and Shengang, Taiwan (*Austruca lactea*) exhibit noticeable differences in claw color, body size, and morphology. Images are taken from the videos we analyze, demonstrating the challenge of limited resolution which our software is able to overcome.

Fiddler crab video data were collected in Thailand in 2023, featuring the species *Austruca annulipes*. Additional videos were sourced from collaborators in Australia and Taiwan, representing *Austruca mjoebergi* and *Austruca lactea*, respectively (Figure 1).^2^ These data sets were chosen to evaluate the algorithm's robustness across varied species and environmental conditions.

The crabs differ slightly in body and claw size^3^ as well as coloration, which can affect visibility and tracking performance. Environmental conditions also vary: some recordings feature high‐contrast mudflats where crabs are easily distinguishable, while others involve more cluttered or low‐contrast backgrounds where crabs blend into their surroundings. These differences introduce natural variation in visibility and scene complexity, allowing us to test the generalizability of our approach across different field conditions.

### 2.2 Algorithm Overview

**FIGURE 2:**
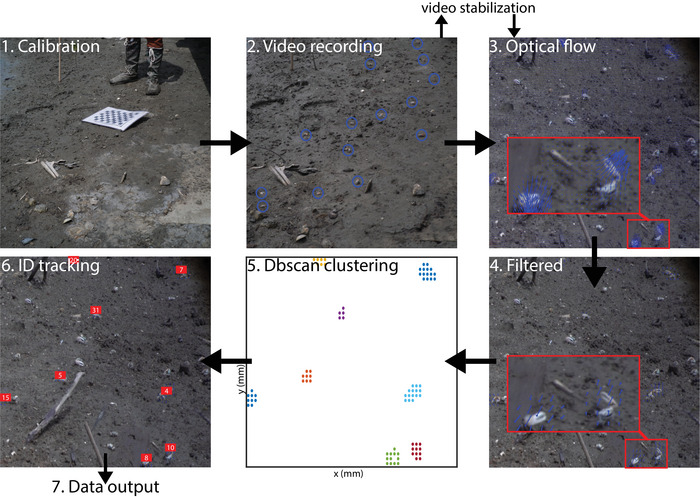
Crab motion tracking workflow. A schematic of the process for obtaining crab claw waving data, including camera calibration, video recording, optical flow, filtering, clustering, and ID tracking. Blue circles in (2) show where the crabs are in the scene.

We developed a motion‐tracking algorithm for identifying individual crabs in field videos by leveraging the strengths of existing optical flow and density‐based clustering methods. The algorithm operates frame by frame, estimating motion, filtering it for significant activity, clustering motion points to identify individual crabs, and tracking them over time. The sections below describe each component of the algorithm in detail (see also Figure 2 for a schematic).

#### 2.2.1 Motion Estimation

We infer velocity fields using the Farneback method [20], which estimates dense optical flow by fitting a polynomial function to the neighborhood around each pixel. It calculates the displacement between two consecutive frames by looking at how the polynomial coefficients change. A key feature of this method is the use of a multilevel pyramid where motion is estimated at different resolutions to make the results more stable. Due to this dense calculation, the Farneback method is robust to noise but can be computationally expensive. For cleaner videos, or when faster processing is more important than maximizing accuracy, sparse optical flow methods can be used instead.

#### 2.2.2 Downsampling and Filtering

After dense motion data are computed, the velocity field resolution is reduced by dividing the frame into a uniform grid of cells (size specified by the user) and then averaging within each. A threshold operation is then applied to replace low magnitude velocities (cutoff specified by the user) with exactly zero, leaving a sparse field of motion vectors that is relatively stable and robust to noise.

#### 2.2.3 Motion Clustering With DBSCAN

To identify clusters in the filtered velocity field that should be treated as compact unified objects, we use the DBSCAN (density‐based spatial clustering of applications with noise) algorithm [21]. This algorithm, unlike many competing methods for clustering, does not require the number of clusters to be specified in advance, and is able to identify clusters of arbitrary shape. These features are especially useful in the context of field recordings, since the number and positions of crabs can vary during and across videos.

DBSCAN works by sequentially aggregating neighboring points (within a given search radius) into a single cluster subject to the constraint that the resulting cluster have at least some minimum size. For our videos, we set the search radius parameter epsilon to 30 pixels, which works well across conditions, though that might need to be revisited in the future for different resolution videos or for crabs that appear much larger or smaller. We set the minimum cluster size parameter, minpts, to two pixels, since crabs appeared small in many of our videos and we wish to err on the side of inclusivity (we can later filter out spurious data based on inferred phase dynamics).

After DBSCAN clustering, we compute the centroid of each cluster, which we use to assign and track IDs. Each cluster in the current frame is matched to the closest cluster in the previous frame using Euclidean distance, and the match is accepted if the distance is within the threshold (maxDistanceThreshold in our code). See the Supplementary Material for more details on parameter choices in our algorithm.

## 3 Results

**FIGURE 3:**
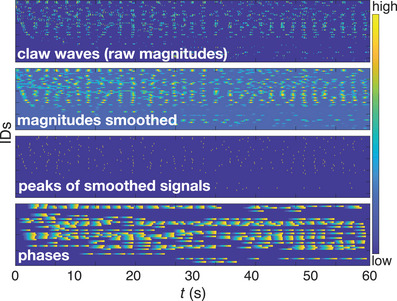
Example of output data and analysis for *A. annulipes*. Data from 50 individuals over the course of 1 min. We show (from top to bottom) how we go from raw data (obtained from our algorithm) to more insightful data. As the initial output can be noisy, we first apply smoothing and then detect the peaks of these smoothed signals (i.e., the moments of highest magnitude). We treat each crab as an oscillator creating these periodic peaks, and thus interpolate between them to get phases over time for each crab during the periods when it is actively waving. Colorbar: In each panel, we display the data in a heatmap, with colors ranging from dark blue (low *R* values) up to yellow (high values). For example, in the top panel brighter colors mean that, at that point in time, an individual had higher claw speed magnitude.

We use the output of our tracking algorithm (filtered velocity field estimation plus clustering) to extract the time of maximum speed for each claw wave, which we treat as phase zero for each cycle. This allows us to then interpolate to obtain phases over time for each individual crab, as shown in Figure 3. Some synchrony is evident in the figure (though analysis of the data is not our focus here): columns of data appear vertically aligned across panels—particularly visible in the top two. We can quantify this synchrony at R=0.46 for this minute of sample data (where R is the Kuramoto order parameter [22]), about double what would be expected by chance.

**FIGURE 4:**
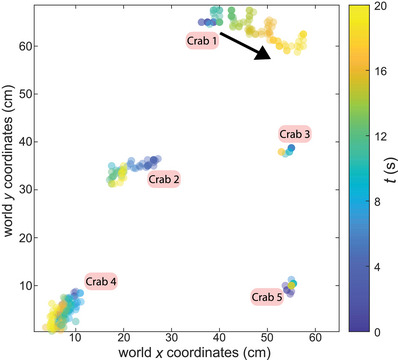
Crab positions over time. Sample data showing raw 2D positions in metric space in a time window of 20 s. We only included IDs with at least one full claw wave within our specified time frame. As such IDs that were not present for a full movement were discarded. Each point represents the center of mass of the crab.

We can also generate trajectory plots to visualize how individual crabs move in space (Figure 4)—highlighting their relatively immobile nature while synchronizing. Even so, occasional translative movements can be observed and may be related to the timing of claw waves and how one crab's motion or position influences the phases of others nearby.

### 3.1 Accuracy

To quantify accuracy, the algorithm's crab detection across several videos is compared to human crab detection (as assessed by one of the coauthors). Table 1 summarizes these results. For the *A. annulipes* crabs, it appears that the algorithm generally finds fewer crabs than a human, whereas the reverse was true for the other two species (*A. mjoebergi* and *A. lactea*). This may be due to the small apparent size and low contrast of the crabs in videos of *annulipes*. Erring on the side of excess crabs detection should generally be preferred, since we should often be able to eliminate spurious IDs based on waving dynamics (or lack thereof).

**TABLE 1:** Algorithm accuracy.

Video	Frame count	Number detected by algorithm $N_{\mathrm{alg}}$	Number detected by human $N_{\mathrm{hum}}$	Agreement accuracy	Relative error	Net error
Thailand C0002	1835	23	27	85%	15%	−4
Thailand C0004	1335	50	53	94%	5.7%	−3
Taiwan	240	15	13	85%	15%	2
Australia MAH00592	1813	33	29	86%	14%	4

*Note*: Summary of the algorithm's crab detection accuracy across multiple field videos, as compared to manual human annotation. Agreement accuracy reflects how closely the algorithm matches human annotations, while relative and absolute errors quantify the magnitude of disagreement. “Net error” is defined as $N_{\mathrm{alg}}-N_{\mathrm{hum}}$, where $N_{\mathrm{alg}}$ is the number of crabs identified by the algorithm and $N_{\mathrm{hum}}$ is the number of crabs identified by a human watching the same video (taken to be the ground truth). “Relative error” is calculated as $\left|\left(N_{\mathrm{alg}}-N_{\mathrm{hum}}\right)/N_{\mathrm{hum}}\right|\times 100\%$, and “Agreement accuracy” is 100%−Relativeerror.

Concretely, our algorithm achieves agreement accuracies ranging from 85% to 94%, with absolute errors between two and four individuals, and relative error consistently below 16%. Accuracy and results are very likely to improve both with finer parameter tuning and under more controlled experimental conditions. For example, recording crabs in an environment with a stable camera, uniform lighting, and homogeneous background would help isolate relevant motion and improve detection. In addition, using a defined arena where the crabs remain within the frame would likely make tracking and behavior classification more reliable. These results demonstrate, however, that the algorithm can reliably recover estimates of group size even in visually challenging scenes. Notably, the highest agreement accuracy was observed in the Thailand C0004 video, which also had the largest number of individuals, suggesting that the algorithm scales well with group size.

Regarding automatic recognition of waves, for the data shown in Figure 3, human annotators recorded 609 total waves by 34 total crabs (with around 15 typically active at any time). The algorithm missed two claw waves for one crab and one claw wave for four other crabs, yielding a net accuracy of around 98%.

### 3.2 Algorithm Speed

To evaluate the runtime performance of this method, its speed on field‐recorded videos of fiddler crabs captured in complex natural environments was compared to that of existing tracking algorithms on benchmark videos recorded in controlled laboratory settings. While the comparison involves very different recording conditions, it provides a general point of reference for assessing relative computational efficiency.

In Table 2, we show average runtimes for both our crab videos and the videos used by Sridhar et al.  in their 2019 Tracktor paper [12] (tracking termites and zebrafish). In each case, the tracking load was comparable—a similar number of animals were visible in each video.

**TABLE 2:** Runtime analysis summary.

Video	Source	Frames	Number of individuals	Tracktor runtime	ToxTrac runtime	Our algorithm runtime
Thailand C0002 ‐ field	—	1200	6	—	—	05:25.9
Australia 00592 ‐ field	—	1200	6/7∗	—	—	17:03.7
Termite ‐ lab	Tracktor	1200	8	04:09.7	00:12.0	—
Zebrafish ‐ lab	Tracktor	1200	5	01:57.2	00:13.5	—

*Note*: We report runtimes for tracking all individuals in four different videos (runtimes averaged over 10 realizations). Note that videos using Tracktor algorithm were lab videos from [12], not field videos. Time format is mm:ss.s; default/recommended parameters were used for each algorithm; tests performed under Windows 10 with an 11th Gen Intel Core i7‐1165G7 CPU @ 2.80 GHz and 16 GB RAM.

## 4 Discussion

Our algorithm is clearly considerably slower than ToxTrac and somewhat slower than Tracktor. This is likely due to the overhead of dense optical flow estimation and DBSCAN clustering in every frame, which are more computationally expensive than the background subtraction and blob segmentation used by ToxTrac [11] or the contour detection and k‐means clustering used by Tracktor [12]. Because we analyze all motion across all frames, we capture subtle but meaningful behaviors that can be missed by faster methods focused on centroid tracking or blob detection. Due to these algorithmic differences, the runtime comparisons are not entirely relevant.

As shown in Table 2, the proposed algorithm processed 1200 frames of the Thailand field video in 5 min and 26 s and the more visually complex Australian field video in just over 17 min. While these times are longer than those of Tracktor or ToxTrac—which can process lab videos in under 5 min—the field videos contain significantly more visual clutter and lack the controlled conditions that enable fast centroid‐based tracking. Thus, while the comparison highlights a speed gap, it also underscores the robustness and adaptability of this method in real‐world, uncontrolled environments.

It is possible that the algorithm accuracy could be improved if videos were captured at a higher frame rate. All of our videos have a frame rate of 30 frames per second which may be insufficient to fully capture the rapid and subtle motions of crab claw waving. With more motion information from a higher frame rate, the optical flow estimates would be more precise, especially for fast or small movements that are easily missed. This of course would come at a cost: processing of more frames would mean a linear increase in the computational cost and thus the runtime. It is also likely that obtaining higher resolution videos would improve the accuracy of our algorithm. Currently the fast‐moving claws—the key objects being tracked—occupy only about 15 pixels,^4^ making it easy to miss their motion when spatially averaging estimated velocity fields.

Beyond resolution limitations, optical flow can be influenced by motion parallax, whereby objects closer to the camera appear to move more quickly. Although no correction was applied in this study, positioning a calibration pattern in the field of view should allow for parallax compensation. Also, an orthogonal overhead camera setup (e.g., from a drone) can be used to drastically reduce parallax effects.

The fact that our code works quite well, despite difficult and highly variable conditions in the field, suggests that tracking crab behavior in the lab (with homogenous backgrounds, fixed group sizes, and no spurious movements) should be relatively straightforward. If and when such setups become established, may be there is even scope for neural recordings [23], attempting to reveal the neural underpinnings of sync.

We also see an opportunity for field experiments involving the development and deployment of robotic crabs, which researchers could leverage to perform specific manipulations [24, 25] and interventional experiments.

### 4.1 Limitations

A key limitation of our method is its sensitivity to environmental noise (e.g., foliage movement, ripples in water, or a shaking camera). Very noisy frames can result in essentially random flow vectors, which may be clustered and assigned spurious IDs.

While motion thresholds and the downsampling of dense optical flow can reduce the impact of random perturbations, additional filtering may be necessary. In some cases, for example, motion can be masked by excluding specific regions of the frame based on color or position. However, such strategies are environment‐dependent and may not always be feasible. Manual removal of false‐positive IDs after tracking may still be required.

Due to the frame‐by‐frame nature of our approach and the motion thresholding, gaps can occur in the detection of continuous movement, especially during slower claw waves. If a claw's movement between two consecutive frames falls below the threshold, it may be missed, creating small discontinuities in the recorded motion. Nonetheless, this is not a significant issue, as the resulting time series can be smoothed to recover the complete motion (see Figure 3). These gaps are a known trade‐off of the frame‐by‐frame method, which is essential for reliably detecting the initiation of claw‐waving events.

When crabs are very close to each other in a scene, their flow vectors can overlap and cause their IDs to be switched or merged. Changing the DBSCAN epsilon parameter may help separate these crabs, but its effectiveness depends on the scene, how clustered the crabs are, and how large the crabs are in pixels. Crabs that are very close to the camera or much larger in size may also be assigned two IDs. Similarly, if a crab leaves and reenters the frame, its ID may not be correctly reassigned. In general, as the group sizes we have observed are not as large as some fish schools or bird flocks, and given that crab translational motion is infrequent, this is unlikely to cause practical problems.

The observation that crab translational motion is infrequent may also be influenced by the behavioral tendencies of the genus studied, and may not generalize to all fiddler crab species. In addition, while the species analyzed primarily waves while stationary, other fiddler crab species may move more during or between waves. We expect, though, that our tool can be adapted to more mobile species through parameter tuning.

Finally, our method can be computationally costly, especially in very noisy or dense scenes. Using Farneback optical flow requires large velocity field matrices for each frame, increasing processing time and computational cost. As the number of crabs, their movements, and visual complexity of the scene increase, more time and memory are required.

## 5 Conclusions

We have shown that our algorithm can successfully track groups of crabs in complex natural environments. The ability to extract motion traces, including claw movements, directly from real‐world footage opens new possibilities for studying group coordination and behavioral synchrony in the wild.

Fiddler crabs, in particular, represent a compelling case for this approach [26]. They are found in diverse environments around the world, making them an accessible and ecologically relevant model organism for synchrony research. We hope that this work ultimately leads to new, data‐validated models for how, and why, organisms in nature synchronize.

## Author Contributions

H.K. wrote all software and drafted the initial manuscript. D.M.A. and G.A. obtained funding. G.A. conceived of the research project and performed the fieldwork. All authors contributed to the ideas involved in the project and the final version of the manuscript.

## Conflicts of Interest

The authors declare no conflicts of interest.

## Supporting information



Supplementary Materials: nyas70213‐sup‐0001‐SuppMat.pdf

## Data Availability

The code is available in MATLAB (MathWorks Inc., Natick, MA, USA) or Python (Python Software Foundation, 2018). All code is available on GitHub with the identifier tracking‐for‐synchrony https://github.com/hibakhatib/tracking‐for‐synchrony.

## Footnotes

1 Here, we use the term “synchrony” to refer to temporal alignment of signals. This can take the form of perfect alignment in phase or other forms of temporal order (e.g., with fixed phase lags between the individuals).

2 Note that we do not know the exact details of how these recordings took place (i.e., distance, camera tilt, etc).

3 Mean carapace breadth is around 13 mm for *A. annulipes* and *A. lactea*, and around 11 mm for *A. mjoebergi*.

4 Note that this is far less than the recommended size of tracked objects in other systems like Tracktor.
