# Supplementary material for: Tracking Tool for Fiddler Crabs in Natural Settings to Promote a Model Organism for Synchrony
Source: Ann N Y Acad Sci. 2026 Mar 24;1557(1):e70213. doi: 10.1111/nyas.70213 (PMC13013735; doi:10.1111/nyas.70213)
Supplement: Supplementary file 1 — Supplementary Materials: nyas70213‐sup‐0001‐SuppMat.pdf [file NYAS-1557-0-s001.pdf]

SUPPLEMENTARY MATERIAL FOR: "A TRACKING TOOL FOR FIDDLER CRAB CLAW WAVING IN NATURAL SETTINGS TO PROMOTE A NEW MODEL ORGANISM OF SYNCHRONY" BY HIBA KHATIB, DANIEL M. ABRAMS AND GUY AMICHAY

Additional supplementary material may be found in the online version of the article at the publisher's website.

1 | SOFTWARE

All Matlab code described in this manuscript was developed and tested using MATLAB version 2024b, with the Computer Vision Toolbox (version 24.2) and the Statistics and Machine Learning Toolbox (version 24.2). For Python, it was developed and tested using OpenCV (4.12.0), scikit-learn (1.6.1), NumPy (2.0.2), SciPy (1.16.1), matplotlib (3.10.1), and the standard math module.

2 | PARAMETERS FOR THRESHOLDING AND MOTION FILTERING

Beyond those mentioned in the main text, there are additional important parameters to be considered such as the `grid_size`, `minMagnitude` and `maxMagnitude`, and the `maxDistanceThreshold`. The `grid_size` is relevant to the downsampling of the optical flow vectors and the magnitude and distance parameters are relevant to clustering and ID assignment across frames. There are also flag parameters for indicating if the user wants to create and save motion and position data, `saveData`, and if they want to create a visualization of the identified crabs `createViz` as well as the scale of the vectors of motion being drawn in the visualization `vectorScale`. Creating visualizations can be helpful while searching for optimal parameters but can and should be disabled to minimize computational cost. Table 2 provides a detailed summary of all parameters used in our algorithm, including where each parameter is applied, its role in the pipeline, and practical guidelines for choosing appropriate values.

Two optical flow parameters may be necessary to change based on field conditions: the Gaussian filter size and the neighborhood size. The Gaussian filter size sets the spatial scale for averaging after flow inference; for a windier or noisier video, increasing it makes the algorithm less sensitive to noise from trees or other objects waving in the scene. We used 15x15 pixels as the default size, though we found that increasing it to 50x50 or 70x70 helped under very windy conditions. The neighborhood size parameter sets the spatial scale for polynomial expansion in the Farneback method. Larger neighborhood sizes effectively average the motion over more pixels, making the estimation more robust to noise and slightly faster to compute, but at the cost of lower spatial resolution. We used 5 pixels as the default neighborhood size, and found that increasing it to 7 or 9 pixels helped with noisier videos.

3 | VIDEO PREPROCESSING

Video stabilization methods can be used to minimize video noise caused by camera shake as a preprocessing step. Although this does not significantly reduce overall runtime, it can reduce the time required for manual post-processing by lowering the number of spurious motion detections. We applied stabilization to a 1200-frame segment of the Thailand C0004 video, which exhibited noticeable jitter (see Fig. 1). Stabilization was performed using FFmpeg and the vid.stab library with three different parameter settings representing light, medium, and strong stabilization strengths. For each setting, stabilization was performed in two steps. First, motion detection was performed using vidstabdetect with shakiness and accuracy parameters adjusted for each level (see Table 1). This step outputs a transformation file describing estimated camera movement. In the second step, we applied the stabilization using vidstabtransform with increasing values of smoothing and zoom.

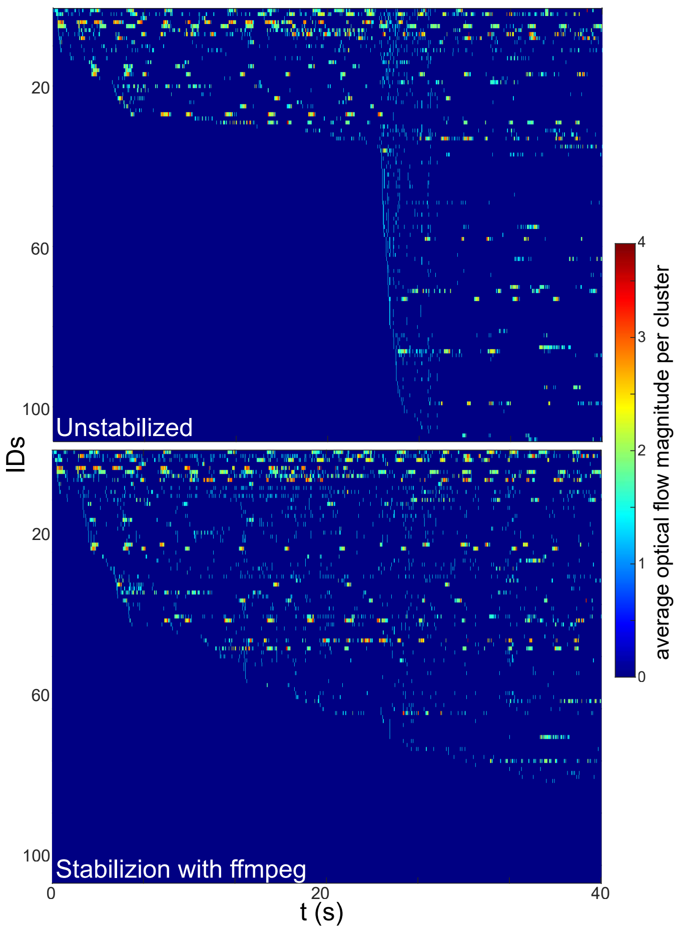

FIGURE 1 Video stabilization effect. Comparison of IDs detected in an unstabilized video (top) and after applying light stabilization using FFmpeg and the vid.stab library (bottom). In the unstabilized condition, many IDs appear suddenly and persist briefly due to noise from camera jitter, especially between frames 700-800. These spurious detections lead to cluttered data and require manual post-processing. After stabilization, noisy detections are significantly reduced, and IDs exhibit more continuous and consistent trajectories, improving data quality and minimizing false positives. Each row represents an ID, and each column is a frame in the video. Color indicates the magnitude of motion detected for that ID in each frame, with warmer colors (yellow/red) showing stronger motion and cooler colors (blue) indicating little or no motion.

Method	Shakiness	Accuracy	Smoothing	Zoom	Total IDs	Total Runtime	Detect/Transform/Algorithm
Unstabilized	-	-	-	-	108	13:53.0	-
FFmpeg - low	3	8	5	0	83	15:14.9	00:23.4/00:51.9/13:59.7
FFmpeg - mid	5	10	15	2	84	15:12.0	00:30.3/00:52.2/13:49.5
FFmpeg - high	10	15	30	5	84	25:28.5	00:21.4/00:35.3/24:31.8

TABLE 1 Stabilization parameters and runtime summary. Stabilization was applied at three levels (low, medium, and high) using vid.stab with increasing values for shakiness, accuracy, smoothing, and zoom. While stabilization did not significantly reduce total runtime, it lowered the number of noisy IDs before post-processing. Runtime is shown in minutes and seconds (mm:ss.s), broken down into stabilization detection, transformation, and main tracking stages. Medium stabilization yielded a good balance between improved data quality and minimal additional processing time.

Parameter	Category	Where used	Purpose	Rules of Thumb
FilterSize	Optical flow	opticalFlowFarneback()	Size of the filter window used to smooth the optical flow estimates; larger values smooth more but may blur small motions.	Default value: 15. Very windy frames with camera shaking: 50, 70 .
NeighborhoodSize	Optical flow	opticalFlowFarneback()	Size of neighborhood for polynomial expansion; affects how local motion is modeled. Smaller = finer details, larger = smoother motion.	Default value: 5. Dense crabs: 7, 9.
gridSize	Flow preprocessing	processOpticalflow()	Defines size of grid cells used to spatially divide motion vectors before clustering. Controls spatial resolution of motion grouping. Used for downsampling dense flow data.	Default value: 25 Changing filterSize is usually enough. This can average out overall motion and you will need to adjust your minMagnitude.
minMagnitude	Flow filtering	processOpticalflow()	Minimum flow vector magnitude threshold; filters out noisy motions like camera shakiness.	Start with: 1.2, 1.3, 1.35. Set the motion threshold based on the median motion values of the correctly identified crabs.
maxMagnitude	Flow filtering	processOpticalflow()	Maximum flow vector magnitude; removes unrealistically large optical flow vectors caused by noise or errors.	Start with: 5. Set based on the median of noisy ids.
maxDistanceThreshold	Cluster matching	processClusters()	Maximum allowed distance between two cluster centroids to associate them as the same crab across frames. Controls tracking sensitivity.	Default value: 80. Fairly generalizable unless crabs are very densely populated; decrease by 2-5 if so.
dbscanEpsilon	Dbscan clustering	processClusters()	Maximum distance between two motion points to be considered neighbors. Controls tightness of motion clusters.	Default value: 30. Fairly generalizable unless crabs are very densely populated; decrease by 2-5 if so.
dbscanMinpts	Dbscan clustering	processClusters()	Minimum number of points needed to form a valid cluster. Controls robustness against noise; higher = more conservative.	Default value: 2. If crabs are large and close to camera, minimally increase. Otherwise, keep at 2 to avoid missing small or farther from camera crabs.

TABLE 2 **Parameter Summary**. Detailed summary of parameters that user can fine tune. The table details the parameter usage context, its purpose, and practical rules of thumb for choosing parameter values. These parameter and method names refer to the MATLAB implementation, but they closely correspond to those used in the Python version.
